# Chemokine Receptor CXCR3 Correlates with Decreased M2 Macrophage Infiltration and Favorable Prognosis in Gastric Cancer

**DOI:** 10.1155/2019/6832867

**Published:** 2019-05-21

**Authors:** Fangfang Chen, Jingping Yuan, Honglin Yan, Huan Liu, Shuai Yin

**Affiliations:** ^1^Department of Pathology, Renmin Hospital of Wuhan University, Wuhan 430060, Hubei, China; ^2^Department of Pathology, Zhongnan Hospital of Wuhan University, Wuhan 430071, Hubei, China

## Abstract

**Aim:**

The aim of this study was to explore the correlation of chemokine receptor CXCR3 with M2 macrophage infiltration, various clinicopathological features, and prognosis in patients diagnosed with gastric cancer (GC).

**Methods:**

Expression of CXCR3 protein and M2 macrophage was evaluated in 156 GC patients and corresponding paracancerous tissues by immunohistochemical (IHC) analysis.

**Results:**

In our study, 59 (37.82%) showed high expression of CXCR3 protein in 156 GC tissues. Expression of CXCR3 protein was significantly increased in tumor tissues compared with corresponding paracancerous tissues (*P* < 0.001). Overexpression of CXCR3 protein correlated with decreased M2 macrophage infiltration (*P* = 0.001). By analyzing the association between expression of CXCR3 protein and clinicopathological factors of GC patients, we found that high level of CXCR3 protein was significantly correlated with better differentiation (*P* =0.017), I/II TNM stage (*P* = 0.02), and smaller invasion depth (*P* = 0.003). Moreover, we found through Kaplan-Meier analysis and log-rank test that GC patients with high expression of CXCR3 protein and low M2 macrophage infiltration had better overall survival (OS) and low mortality rate (*P* < 0.001 and* P* = 0.024, respectively). The multivariate survival analysis showed that high expression of CXCR3 protein could serve as a favorable independent biomarker for prognosis in GC patients [hazard ratio (HR): 0.342 (0.204-0.571);* P* < 0.001].

**Conclusion:**

Our study indicates that overexpression of CXCR3 protein in GC is associated with decreased M2 macrophage infiltration and improved OS and thus can be further exploited as a biomarker in GC.

## 1. Introduction

Gastric cancer (GC) is by far the fifth most common malignant tumors all over the world and has high mortality: it was the third-leading cause of cancer-related deaths worldwide [1]. The prognosis of the GC is significantly correlated with the tumor-associated immune cells in tumor immune microenvironment [2]. Tumor-associated immune cells such as natural killer (NK) cells, dendritic (DC) cells, helper T1 (Th1) cells, and cytotoxic CD8 cells in the tumor microenvironment are generally correlated with favorable outcome [3–6]. In contrast, infiltration of B cells, regulatory T (Tregs) cells, and Th2 and Th17 cells promotes tumor development and correlates with a poor prognosis [7–9]. Tumor-associated macrophages (TAMs), expressing M1 and M2 macrophage phenotypes, also play a crucial role during the tumorigenesis and development of GC [10]. The M1 macrophages can induce apoptosis, reduce proliferation of tumor cells, and inhibit the development of neovascularization. On the contrary, M2 macrophages can promote both tumor growth and metastasis and predict a poor prognosis [11–13].

Chemokine receptors, which are a superfamily of G-protein-coupled receptors, involve in numerous biological processes, including cell adhesion and migration, by binding to their ligands [14]. Furthermore, it has been proved that chemokine receptors participate in tumor growth and progression, such as angiogenesis and metastatic dissemination [15]. CXCR3 belongs to ELR-negative CXC chemokine receptors. A large number of studies indicated that CXCR3 performs different roles in the tumorigenesis and progression of various cancers. For instance, Shen and his colleagues proved that aberrant expression of CXCR3 might suppress the proliferation and invasion of prostate cancer [16]. Murakami et al. showed that expression of CXCR3 could promote metastasis and predict a poor prognosis in colorectal cancer [17]. And Rezakhaniha et al. have demonstrated that high expression of CXCR3 was significantly related with tumor stage and shorter overall survival in clear cell renal carcinoma [18].

The chemokine receptor CXCR3 has also been known for its indispensable role in tumor immune microenvironment, which could promote the migration, activation, and differentiation of some tumor-associated immune cells [19–21]. In our previous studies, we have demonstrated that the expression of CXCR3 protein in GC was significantly higher than that in adjacent paracancerous tissues and associated with a better prognosis [22]. Furthermore, high expression of CXCR3 protein was closely correlated with the increased recruitment of CD4^+^, CD8^+^ tumor infiltrating lymphocytes (TILs), and dendritic cells, which may partly explain the favorable prognosis in GC [22]. It has been reported that CXCR3 deficiency correlates with infiltration of macrophage M2 and enhances tumor progression in breast cancer [23]. However, the report about the role of CXCR3 in GC has not been widely reported, and the reason why high CXCR3 protein expression correlates with a longer survival and lower mortality rate in GC remains to be determined.

In this study, we evaluated the association of CXCR3 protein expression with macrophage infiltration, clinicopathologic characteristics, and prognosis in GC patients. Furthermore, we investigated the potential of overexpression of CXCR3 protein as an independent indicator of prognosis in GC patients.

## 2. Material and Methods

### 2.1. Patient Tissue Samples

A total of 156 formalin-fixed, paraffin-embedded GC tissue samples and their adjacent (≥5 cm) nonneoplastic tissue specimens (considered as the normal group) were obtained from the patients diagnosed with GC through histopathologic evaluation on gastroscopic biopsy or surgical tissue specimens. All the patients underwent surgical treatment at Zhongnan Hospital of Wuhan University in the period from July 2008 to December 2013. There were no any previous chemotherapies, radiotherapies, or other treatments before surgery in these patients. The study was approved by the Ethics Committee of Zhongnan Hospital of Wuhan University and the Ethics Committee of Renmin Hospital of Wuhan University.

The patients were composed of 114 males and 42 females with a median age of 58 (age range, 24–85) years. Among the 156 cases, 69 (44.23%) were classified into intestinal GC and 87 (55.77%) were defined with diffuse GC on the basis of Lauren's classification [24]. In accordance with the AJCC TNM stage classification system [25], 44 (28.21%) patients were classified as stages I and II and 112 (71.79%) as stages III and IV. Moreover, the treatment of stage I and II patients is same, so is the treatment of stages III and IV. Other basic clinicopathological characteristics, including age, gender, histological differentiation, tumor diameter, tumor infiltration depth, regional lymph node involvement, and histological differentiation for each patient, were presented in Table 3.

Follow-up started on the date of operation and ended in December 2015 with a median follow-up time of 21.5 months (ranged from 4.5 to 88.5 months). OS was calculated as the period from the date of operation to the end of follow-up or death. We followed-up all the patients by telephone interviews or outpatient clinic visits. At the end of follow-up, 73 patients (46.79%) were alive and 83 (53.21%) died of GC.

### 2.2. Immunohistochemistry (IHC)

A tissue array was used which included one to three tumor samples from each patient. The tissues were fixed in a 10% formaldehyde solution and then embedded in paraffin. Next, the paraffin tissues were cut into 4 um-thick sections, dried, dewaxed in xylene, and dehydrated in ascending series of ethanol. Subsequently, paraffin sections were rinsed with PBS (3×5 min) and then blocked with 3% hydrogen peroxide at room temperature for endogenous peroxidase ablation for 10 min. Antigen retrieval was conducted by microwave heating with citrate buffer (pH 6.0) for 20 min. Then the samples were exposed to normal goat serum at room temperature for 20 min to decrease nonspecific antibody binding. The tissue sections were incubated overnight at 4°C with the primary antibody (anti-CXCR3, 1:200, BAO759, WuHan Boster, Wuhan, China; anti-CD163, Ready-to-use, ZM-0428, Beijing Zhongshan Jinqiao Biological Technology Co., Ltd., Beijing, China). After rinsing in PBS, the tissue sections were incubated with horseradish peroxidase-labeled anti-rabbit antibodies at 37°C for 20 min. Then, the tissue sections were rinsed with PBS for 4 times and then dripped with freshly prepared 3,3-diaminobenzidine (DAB). Microscopically, the staining was terminated when the tissue sections were brown-yellow or brown. Subsequently, all the tissue sections were restained with hematoxylin for about 1 minute. Finally, the slices were dehydrated with ethanol and toluene and then sealed with neutral gum. PBS was used to replace the primary antibody as a negative control.

### 2.3. Evaluation of Immunohistochemical Staining

The slides were viewed via Olympus BX53 (Tokyo, Japan) microscope. IHC staining was evaluated independently by two pathologists under the double blind condition. CXCR3 was mainly expressed in cytoplasm of tumor cells. CXCR3 immunohistochemical staining in tumor cells was evaluated semiquantitatively as follows: (1) staining intensity: 0 (no staining), 1 (weak staining), 2 (moderate staining), and 3 (strong staining); (2) the extent of staining: 0 (≤5%), 1 (6-25%), 2 (26-50%), 3 (51-75%), or 4 (76-100%). Three most representative fields of high magnification (400×) were selected to calculate the final score. The final immunohistochemical score was the product of staining intensity and extent: ≤1 was low expression; ≥2 was high expression.

We used CD163 as a marker to evaluate the number of M2 macrophages. M2 macrophages were predominantly located in cell membranes and cytoplasm. Immunohistochemical staining sections were examined at low magnification (100×), and then the five most representative views of high magnification (400×) were selected to assess the number of CD163-positive macrophages. The two pathologists counted the number of CD163-positive macrophages at each high magnification and then averaged them separately. If the two numbers differ by more than 10 cells per high magnification, they would be counted again after a week until the differences were below 10 cells. According to the median value of CD163-positive macrophages, the cases were divided into two groups of low density and high density.

### 2.4. Statistical Analysis

SPSS 17.0 software (Chicago, IL, USA) was used to carry out all the statistical analysis. Comparison of CXCR3 protein expression between GC tissues and adjacent paracancerous tissues was evaluated by using Wilcoxon signed-rank tests. Statistical associations of CXCR3 protein expression with clinicopathological features were assessed through the chi-square test. The associations of the expression of CXCR3 protein with M2 macrophages infiltration and other clinicopathological parameters were analyzed with the nonparametric Spearman rank correlation coefficient. The survival curves were disposed by using the Kaplan-Meier method and log-rank test. We performed univariate and multivariate survival analysis through Cox proportional hazard regression model to assess the independent prognostic factors in GC patients. Hazard ratios (HRs) and their 95% confidence intervals (CIs) were calculated for both univariate and multivariate analyses. Two-tailed* p* values of <0.05 were considered statistically significant.

## 3. Results

### 3.1. Expression of CXCR3 in GC Tissues and Paracancerous Tissues

**Figure 1:**
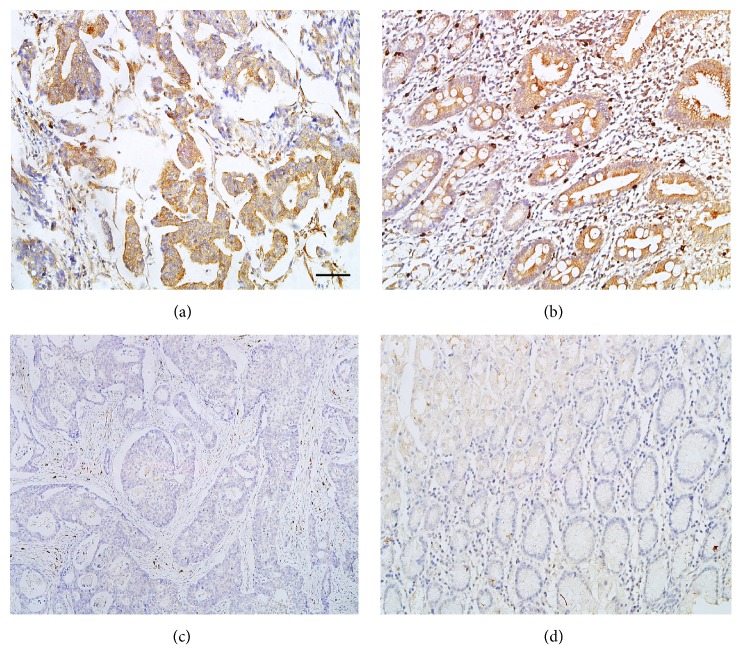
Immunohistochemical staining of CXCR3 in gastric cancer (GC) lesions and their corresponding paracancerous tissues. The staining of CXCR3 protein (brown) was mainly located in the cytoplasm of GC tumor cells: (a) high expression of CXCR3 protein in GC; (b) high expression of CXCR3 protein in nonneoplastic tissues; (c) low expression of CXCR3 protein in GC; (d) low expression of CXCR3 protein in nonneoplastic tissues. Scale bar, 50*μ*m.

To examine the expression level of CXCR3 protein, we performed IHC on 156 GC tissue and the corresponding paracancerous tissue samples. As shown in Figure 1, CXCR3 protein was mainly localized in the cytoplasm of GC cells.  Table 1 showed the results of IHC staining of CXCR3 protein. Of 156 GC samples, 59 (37.82%) showed high expression of CXCR3 protein and 97 (62.18%) showed low expression. In paracancerous tissues, 26 (16.67%) showed high expression of CXCR3 protein, and 130 (83.33%) showed low expression. The chi-square test showed that level of CXCR3 was significantly increased in GC tissues (*P *< 0.001).

### 3.2. Correlation between CXCR3 Protein Expression with M2 Macrophages Infiltration and Clinicopathological Parameters in GC Tissues

**Figure 2:**
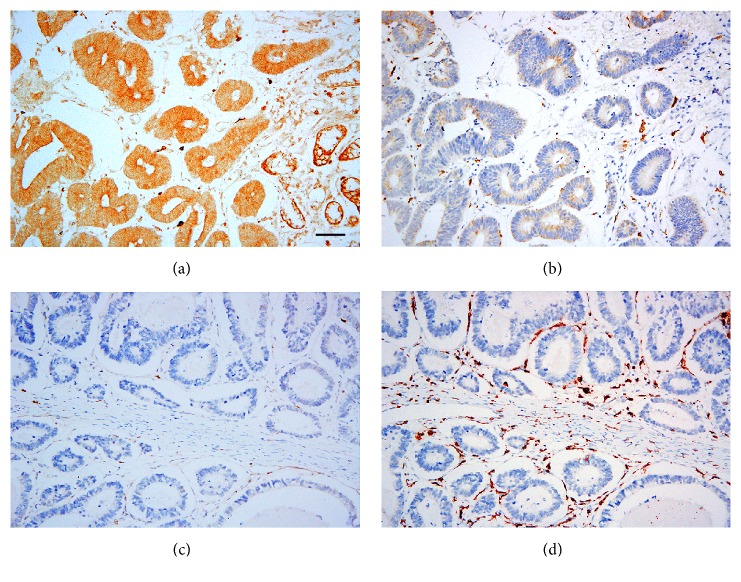
Immunohistochemical staining for CXCR3 and CD163 (M2 macrophage) in GC lesions: high expression of CXCR3 protein (a) with less M2 macrophage infiltration (b); low expression of CXCR3 protein (c) with more M2 macrophage infiltration (d). Scale bar, 50*μ*m.

As shown in Figure 2, immunohistochemical staining of CD163 revealed diffuse staining in the membranes and cytoplasm of M2 macrophages.  Table 2 showed that there was no significant association between expression of CXCR3 protein and M2 macrophages infiltration in adjacent normal tissues (*P *= 0.767). In contrast, high expression of CXCR3 protein was inversely associated with M2 macrophages infiltration in GC tissues (r = -0.286,* P* = 0.001). Furthermore, low expression of CXCR3 protein was detected in 62.18% (97/156) of GC tissues, which was significantly associated with poorer differentiation (*P* = 0.017), more advanced (III/IV) TNM stage (*P* = 0.02), and deeper invasion depth (*P* = 0.003), but not with other examined clinicopathological parameters, including gender (*P* = 0.483), age (*P* = 0.303), Ki67 expression (*P* = 0.173), tumor diameter (*P* = 0.248), lymph node metastasis (*P* = 0.143), or Lauren's classification (*P* = 0.716) in tumor samples.

### 3.3. Correlation Analysis of the Overall Survival Rate with the Expression of CXCR3 Protein, M2 Macrophages Infiltration, and Other Parameters

**Figure 3:**
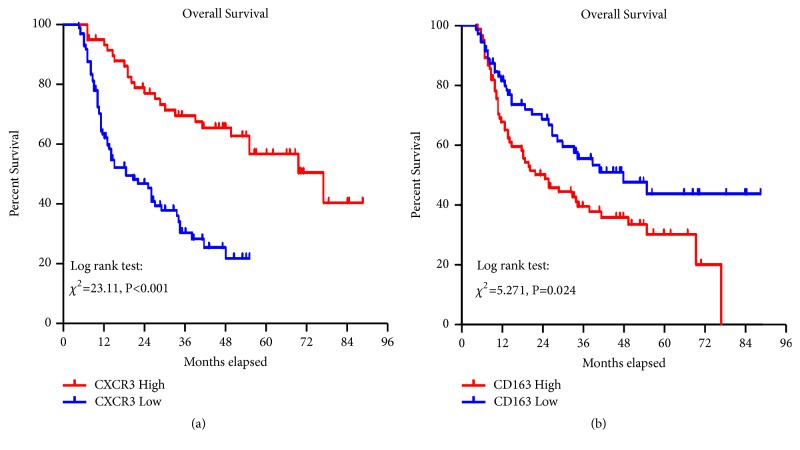
Correlation analysis of CXCR3 and M2 macrophage expression levels with OS: (a) patients with high expression of CXCR3 protein in tumors had longer OS (log-rank test,* P*<0.001); (b) low M2 macrophage infiltration in tumors correlates with longer OS (log-rank test,* P*=0.024).

As shown in Figure 3(a), OS of GC patients with high CXCR3 protein expression was significantly improved (*P* < 0.001). Similarly, low expression of M2 macrophages also correlated to a better prognosis in GC patients (Figure 3(b),* P* = 0.024). Univariate analyses of predictive factors for OS in GC patients were performed by Cox proportional hazards regression model (Table 4). In this analysis, both TNM stage and tumor infiltration depth were significantly associated with OS of GC patients (*P* = 0.004 and* P *= 0.005, respectively). However, Lauren's classification, tumor diameter, lymph node metastasis, differentiation, and age had no significant correlation with OS in GC patients (*P* > 0.05) (Table 4). Moreover, a multivariate Cox proportional hazard model was performed to identify which of the above factors were independent prognostic factors for GC. The results showed that the expression of CXCR3 protein could serve as an independent prognostic parameter for OS of GC patients [hazard ratio (HR): 0.342 (0.204-0.571);* P* < 0.001; Table 4]. Concurrently, depth of invasion was also an independent prognostic factor for GC.

## 4. Discussion

Although some prognostic biomarkers have been identified, more new biomarkers are still needed to elucidate the GC progression and predict the treatment responses and prognosis of GC patients. In this study, we reported that the overexpression of CXCR3 protein in GC is associated with decreased M2 macrophage infiltration and a relatively better prognosis. This is the first study reporting the clinical potential of the association between CXCR3 and M2 macrophages in GC.

CXCR3, a seven-transmembrane G-protein-coupled receptor (GPCR), is considered a putative receptor for the inducible chemokine ligands CXCL9/MIG, CXCL10/IP10, CXCL11/ITAC/IP9, CXCL4/PF4, and its variant CXCL4L1/PF4V1. In tumor tissue, CXCR3 has been found to be expressed in the cancer cells, peritumoral stromal cells, vascellum, and recruited leucocytes, which could regulate tumor growth, migration, invasion, angiogenesis, and immunity, thus directly or indirectly participating in tumor progression. In the present study, we found that CXCR3 protein was primarily located in the cytoplasm of tumor cells in GC tissues. The relative expression of CXCR3 protein in GC tissues was significantly higher than that in corresponding paracancerous tissues, and the high expression of CXCR3 protein was inversely associated with more malignant phenotypes including poor tumor differentiation, TNM stage, and depth of tumor invasion. Such results are similar to those previously reported [26, 27]. Furthermore, by the multivariate analysis, we found that overexpression of CXCR3 protein in GC tissues could be an independent better prognostic factor for GC patients. Consistent with this result, it has been showed that high expression of CXCR3 was associated with a favorable prognosis in clear cell renal carcinoma and prostate cancer [28, 29]. Therefore, these data suggested that CXCR3 has the potential of being a favorable prognostic marker in GC.

M2 macrophages are considered to be essential immune cells that play a critical role in tumor growth, angiogenesis, and metastasis. Previous studies indicated that higher density of M2 macrophages in tumor were closely associate with tumor progression and poor prognosis [30]. Moreover, high density of M2 macrophages was correlated with a poor prognosis in patients with GC [10]. CXCR3 has long been known to promote the migration, activation, and differentiation of some immune cells in tumor microenvironment and has been shown to play an important role in neoplastic diseases. It has been demonstrated that CXCR3 deficiency showed increased proportion of Th2 cells, resulting in high level of IL-4 [31]. Moreover, anti-inflammatory mediators such as IL-4 could induce macrophage M2 polarization [32, 33]. Furthermore, Steve et al. revealed that CXCR3 deficiency displayed increased IL-4 production and M2 polarization in the tumors [23]. Thus, it is possible that overexpression of CXCR3 might decrease proportion of Th2 cells and IL-4 level, reducing M2 macrophage infiltration. In our present study, we demonstrated that decreased M2 macrophage infiltration was associated with the overexpression of CXCR3 protein, thus supporting the view that CXCR3 may act as an important role in the progression of GC via suppressing M2 macrophage polarization and promoting antitumor immunity [23]. Moreover, elevated expression of CXCR3 protein in GC tissues also correlated with a more favorable prognosis, which may be contributed at least in part to the low M2 macrophages infiltration. In addition, we have found that high expression of CXCR3 protein was closely correlated with the increased recruitment of CD4^+^, CD8^+^ tumor infiltrating lymphocytes (TILs), and dendritic cells in our previous study [22]. In this study, we have observed that overexpression of CXCR3 protein was closely correlated with decreased M2 macrophage infiltration. Therefore, CXCR3 may associate with less M2 macrophages, greater dendritic cells, CD4^+^, and CD8^+^ TILs infiltration, thereby resulting in an improved OS in GC.

## 5. Conclusion

In conclusion, we demonstrated that CXCR3 was overexpressed in GC patients and inversely associated with poor tumor differentiation, TNM stage, and depth of tumor invasion. Our data also elucidated that high expression of CXCR3 protein correlated with less M2 macrophages infiltration and independently associated with better OS in GC patients, suggesting that CXCR3 may be associated with the infiltration of several types of immune cells in the immune microenvironment of GC, especially with M2 macrophages. Subsequently, such regulation promotes antitumor immunity, thus affecting the prognosis of GC. Therefore, the expression of CXCR3 protein may be further exploited as a potential prognostic marker in GC.

## Figures and Tables

**Table 1:** The expression of CXCR3 protein in gastric cancer and paracancerous tissues.

	High-CXCR3 expression	Low-CXCR3 expression	*P*-value
Gastric cancerous tissue (n = 156)	59 (37.82%)	97 (62.18%)	*0.001*
Paracancerous tissue (n = 156)	26 (16.67%)	130 (83.33%)	

**Table 2:** The expression of CXCR3 protein and CD163 infiltration in paracancerous tissues.

	High-CXCR3 expression	Low-CXCR3 expression	*P*-value
	(n = 26)	(n = 130)	
CD163			
High	17 (17.35%)	81(82.65%)	0.767
Low	9 (15.52%)	49 (84.48%)	

**Table 3:** Correlation analysis of the expression of CXCR3 with M2 macrophage infiltration and the clinicopathologic parameters.

	High-CXCR3 expression	Low-CXCR3 expression	*P* value
	(n = 59)	(n = 97)	
CD163			*0.001*
High	22 (37.29%)	62 (63.92%)	
Low	37 (62.71%)	35 (36.08%)	
Gender			0.483
Male	45 (76.27%)	69 (71.13%)	
Female	14 (23.73%)	28 (28.87%)	
Age			0.303
< 58	33 (55.93%)	46 (47.42%)	
≧58	26 (44.07%)	51 (52.58%)	
Diameter (cm)			0.248
< 5	33 (55.93%)	45 (46.39%)	
≧5	26 (44.07%)	52 (53.61%)	
Ki67			0.173
<10%	9 (15.25%)	8 (8.25%)	
≧10%	50 (84.75%)	89 (91.75%)	
Lymph node metastasis			0.143
No	19 (32.20%)	21 (21.65%)	
Yes	40 (67.80%)	76 (78.35%)	
Differentiation			*0.017*
Well-moderately	21 (35.59%)	18 (18.56%)	
Poorly	38 (64.41%)	79 (81.44%)	
Invasion depth			*0.003*
T1/T2	15 (65.22%)	8 (34.78%)	
T3/T4	44 (33.08%)	89 (66.92%)	
TNM stage			*0.02*
I+II	23 (38.98%)	21 (21.65%)	
III+IV	36 (61.02%)	76 (78.35%)	
Lauren's classification			0.716
Intestinal	25 (42.37%)	44 (45.36%)	
Diffuse	34 (57.63%)	53 (54.64%)	

T1, tumor invades lamina propria, muscularis mucosae, or submucosa; T2, tumor invasion of the muscularis propria; T3, tumor invasion subserosal connective tissue; T4, tumor invasion serosal or adjacent structures.

**Table 4:** Univariate and multivariate analyses of predictive factors for the overall survival in GC patients.

	n	Univariate		Multivariate	
		*P*-value	Hazard ratio, 95% CI	*P*-value	Hazard ratio, 95% CI
CXCR3 expression		*0.001*	0.306 (0.184-0.51)	*0.001*	0.342 (0.204-0.571)
High	59				
Low	97				
M2 macrophage		*0.024*	1.633 (1.068-2.589)		
High	84				
Low	72				
Age		0.093	1.447 (0.94-2.27)		
<58	79				
≥58	77				
Lauren's classification		0.223	1.309 (0.848-2.021)		
Intestinal	69				
Diffuse	87				
Diameter (cm)		0.96	1.011 (0.658-1.554)		
<5	78				
≥5	78				
Invasion depth		*0.004*	3.111 (1.429-6.733)	*0.024*	2.482 (1.130-5.449)
T1/T2	23				
T3/T4	133				
TNM stage		*0.005*	2.113 (1.251-3.569)		
I+II	44				
III+IV	112				
Lymph node metastasis		0.104	1.530 (0.917-2.553)		
No	40				
Yes	106				
Differentiation		0.151	1.455 (0.872-2.428)		
Well-moderately	39				
Poorly	117				

## Data Availability

The data used to support the findings of this study are included within the article.
